# Development of Transgenic Mice Containing an Introduced Stop Codon on the Human Methylmalonyl-CoA Mutase Locus

**DOI:** 10.1371/journal.pone.0044974

**Published:** 2012-09-14

**Authors:** Nicole E. Buck, Harriet Dashnow, James J. Pitt, Leonie R. Wood, Heidi L. Peters

**Affiliations:** 1 Metabolic Research, Murdoch Childrens Research Institute, The University of Melbourne Department of Paediatrics, Parkville, Victoria, Australia; 2 VCGS Pathology, Murdoch Childrens Research Institute, Parkville, Victoria, Australia; Wageningen University, The Netherlands

## Abstract

The mutation R403stop was found in an individual with *mut^0^* methylmalonic aciduria (MMA) which resulted from a single base change of C→T in exon 6 of the methylmalonyl-CoA mutase gene (producing a TGA stop codon). In order to accurately model the human MMA disorder we introduced this mutation onto the human methylmalonyl-CoA mutase locus of a bacterial artificial chromosome. A mouse model was developed using this construct.

The transgene was found to be intact in the mouse model, with 7 copies integrated at a single site in chromosome 3. The phenotype of the hemizygous mouse was unchanged until crossed against a methylmalonyl-CoA mutase knockout mouse. Pups with no endogenous mouse methylmalonyl-CoA mutase and one copy of the transgene became ill and died within 24 hours. This severe phenotype could be partially rescued by the addition of a transgene carrying two copies of the normal human methylmalonyl-CoA mutase locus. The “humanized” mice were smaller than control litter mates and had high levels of methylmalonic acid in their blood and tissues.

This new transgenic MMA stop codon model mimics (at both the phenotypic and genotypic levels) the key features of the human MMA disorder. It will allow the trialing of pharmacological and, cell and gene therapies for the treatment of MMA and other human metabolic disorders caused by stop codon mutations.

## Introduction

Disruption of the conversion of methylmalonyl-CoA to succinyl-CoA results in an increased level of metabolites in tissues and fluids of the body. This inherited disorder, methylmalonic aciduria (MMA), can be caused by stop mutations in the methylmalonyl-CoA mutase gene (*MUT*). Within the first days of life those with the *mut^0^* form of MMA usually exhibit severe metabolic acidosis along with poor feeding, vomiting, lethargy, hypotonia and secondary metabolic disturbances [1], [2].

The majority of mutations in *MUT* are private missense mutations. A study by Lempp *et al*. found that nonsense mutations occurred in 3 out of a possible 29 mutant alleles [3], making novel nonsense mutations the second most frequent type of mutation in this disorder. Molecular studies of our population of mutase deficient MMA individuals identified 4 out of 7 individuals were compound heterozygous for mutations which added a stop codon, thus the full length enzyme subunits of methylmalonyl-CoA mutase were not produced and the enzyme was inactive (4, plus unpublished data). One way to treat these disorders is to reduce the efficiency of translation termination at stop codons and produce some full length protein.

The R403stop mutation was identified in a patient who presented in the neonatal period and was confirmed to have the *mut^0^* form of MMA [4]. We aimed to produce a mouse model which harbors a stop codon mutation in the *MUT* gene for the purpose of trialing compounds for treatment of MMA patients. This work could also be translated to other disorders caused by stop mutations.

We currently have a knockout MMA mouse model [5] and mice carrying the human *MUT* gene in multiple copies [6]. The knockout mouse (‘MMA knockout mouse’, *Mut* ^−/−^) has no mouse methylmalonyl-CoA mutase gene (*Mut*) and recapitulates the symptoms of the human *mut^0^* form of MMA. This knockout mouse can be partially corrected by the normal human *MUT* gene (‘MMA mouse’, *Mut* ^+/+^ *MUT* ^2h+/−^) to produce a rescue mouse model (‘MMA rescue mouse’, *Mut* ^−/−^ *MUT* ^2h+/−^) [6].

This manuscript describes the development and characterization of the first humanized MMA mouse model with a stop codon mutation (‘MMA stop codon mouse’, *Mut* ^+/+^ *MUT* ^stop+/−^) and its partial rescue with a normal human *MUT* gene (‘MMA stop codon rescue mouse’, *Mut* ^−/−^ *MUT* ^2h+/−^ *MUT* ^stop+/−^).

## Results

### Recombination

**Figure 1:**
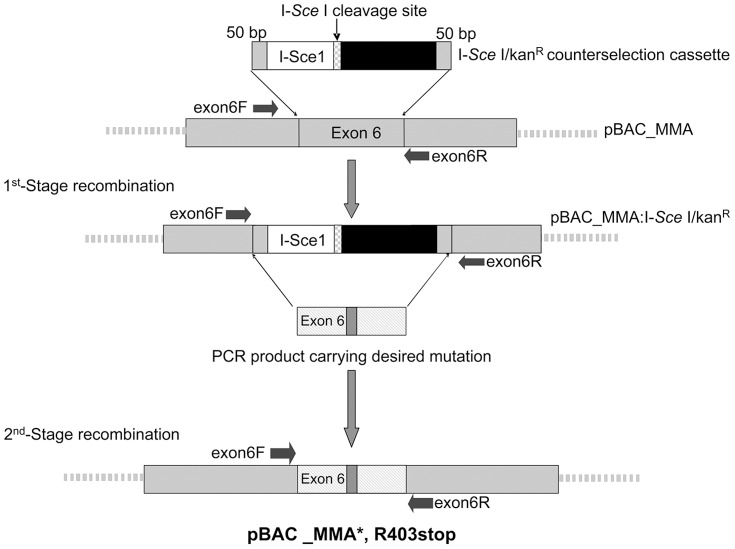
Recombination system for the insertion of the R403stop mutation into the mutase locus of pBAC_MMA. The I-*Sce* I/KanR cassette is flanked with 50 bp regions which are homologous to sequences 50 bp on either side of the site to be targeted in exon 6, and was utilized to introduce the cassette in the first stage. A 255 bp PCR product containing the R403stop mutation was used in the second stage to replace the I-*Sce* I/KanR cassette and produce pBAC_MMA*, R403stop.

**Figure 2:**
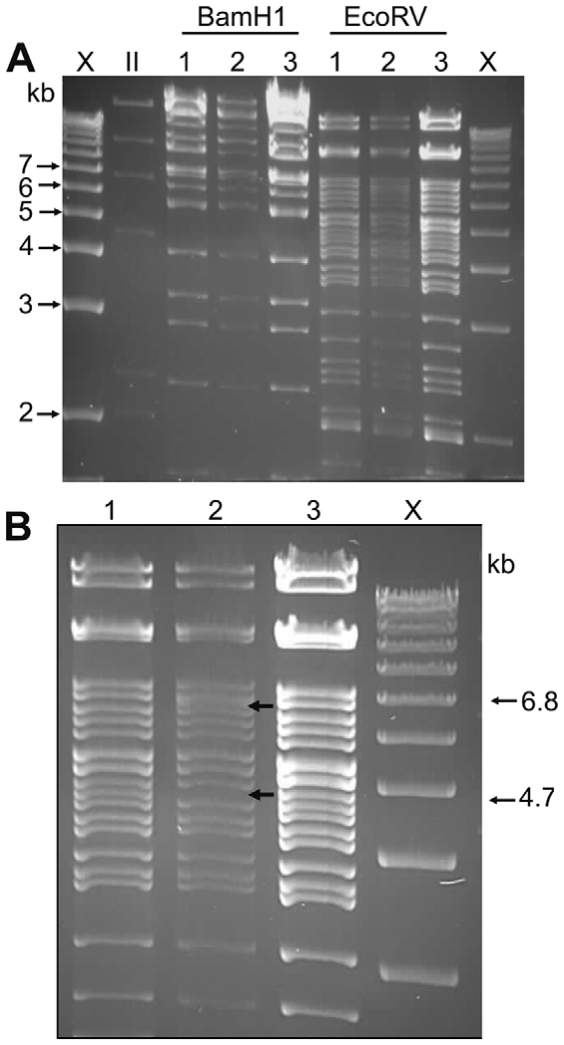
Fine mapping of the unmodified pBAC_MMA. Restriction digestion of 1^st^ stage and 2^nd^ stage recombinant BAC clones to confirm the expected pattern changes and exclude unwanted rearrangements. **A.** *Bam*HI and *Eco*RV restriction digestion. Lane 1. Unmodified pBAC_MMA, Lane 2. 1^st^ stage recombination (insertion of the I-*Sce*I/kanR cassette), Lane 3. pBAC_MMA*, R403stop (2^nd^ stage of recombination). **B.** *Eco*RV restriction digestion of the 1^st^ stage of recombination (Lane 2) showing loss of the 4.7 kb band with the insertion of the I-*Sce*I/kanR cassette (appearance of the expected 6.8 kb band). The restriction digestion pattern of the unmodified pBAC_MMA (Lane 1) and the pBAC_MMA*, R403stop (2^nd^ stage of recombination) (Lane 3) are identical.

The R403stop mutation was inserted into the human mutase locus of bacterial artificial chromosome (BAC), RPCI-11-463L20 (pBAC_MMA) by a two-stage homologous recombination (**Figure 1**). PCR using primers external to the recombination site and restriction digestion with *Taq*I confirmed the introduction of the point mutation in the BAC. Sequencing across the junction of the recombination sites excluded the introduction of additional alterations and confirmed the C→T single base change. Restriction digestion with a panel of enzymes including *EcoR*V and *Bam*HI (**Figure 2**) excluded additional rearrangements in the pBAC_MMA carrying the R403stop mutation and production of pBAC_MMA*, R403stop.

### Microinjection

Three microinjection sessions resulted in two mice positive for the transgene: one male and one female. The microinjection sessions were complicated by low fertility, lysis of embryos after 12 h, and lack of pregnancies following transfers.

### Characterization of the MMA stop codon mouse model transgene

The integrity of the transgene in the MMA stop codon mouse model was determined by PCR of the ends of the BAC and by PCR for exon 3 and exon 12 of the mutase locus. The transgene present in the male founder mouse was confirmed to be intact, however in the female was fragmented. Due to fragmentation of the BAC involving at least the mutase locus, the female mouse was not characterized further and was excluded.

**Figure 3:**
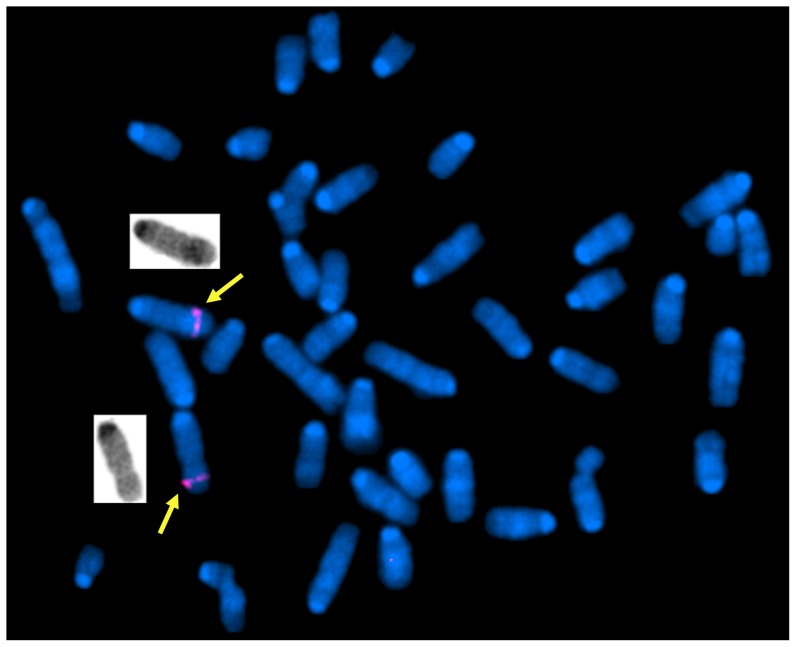
Founder male MMA stop mouse fluorescence *in situ* hybridization studies. The metaphase spread (prepared from fibroblasts) was probed with the intact RPCI-11 463L20 BAC, which was used in the production of these mice. The spread illustrates a cell showing two positive signals (marked with arrows) indicating a single site of integration.

Metaphase FISH of fibroblast culture from the male founder transgenic mouse identified a single site of integration in chromosome 3 (**Figure 3**). Quantitative PCR indicated the founder male mouse had seven copies of the transgene.

### MMA stop codon mouse model breeding

The founder mouse was bred against C57BL/6 mice with transmission of the transgene to more than 12 generations. Normal litter sizes, numbers of male and female mice and Mendelian inheritance of the stop codon transgene occurred.

Metabolite levels in mice which were hemizygous for the stop-codon transgene (*Mut* ^+/+^ *MUT* ^stop+/−^) were similar to control mice (**Table 1**). Control/normal mice were litter mates of the MMA stop codon mice which were homozygous for the mouse *Mut* gene (*Mut* ^+/+^). There was also no differences in mouse growth, development and longevity suggesting the transgene has not caused insertional problems.

**Table 1:** Comparison of metabolite levels in urine and blood of the mouse models.*

Mouse genotype		Urine methylmalonic acid (µmol/mmol creatinine)	C3 propionylcarnitine (µmol/L)
**Control adult (** ***Mut*** **^+/+^)**		119±83 (48)	0.8±0.2 (31)
**MMA stop codon adult (** ***Mut*** **^+/+^** ***MUT*** **^Stop+/−^)**		115±70 (22)	0.8±0.2 (18)
**MMA stop codon rescue adult (** ***Mut^−/−^MUT*** **^Stop+/−^** ***MUT^2h^*** ^**+/−**^ **)**		23400±7800 (25)	12.8±4.3 (9)
**Control pup (** ***Mut*** **^+/+^)**	*1 day prior to birth*	N/A	2.0±0.5 (15)
	*At birth*	123±22 (6)	1.9±0.3 (9)
	*At 16 hours*	109±11 (8)	2.2±0.5 (20)
**MMA Knockout pup (** ***Mut^−/−^*** **)**	*1 day prior to birth*	N/A	10.0±2.5 (4)
	*At birth*	5710±2293 (5)	14.1±1.7 (3)
	*At 16 hours*	20054±6746 (5)	16.9±4.7 (10)
**MMA Knockout stop codon pup (** ***Mut^−/−^MUT*** **^Stop+/−^)**	*1 day prior to birth*	N/A	11.2±3.5 (6)
	*At birth*	3208±1045 (3)	14.7±3.0 (3)
	*At 16 hours*	N/A	17.7±3.0 (3)
**MMA stop codon rescue pup (** ***Mut^−/−^MUT*** **^Stop+/−^** ***MUT^2h^*** ^**+/−**^ **)**	*1 day prior to birth*	N/A	2.3±0.3 (2)
	*At birth*	1406±94 (2)	4.5±1.0 (2)
	*At 16 hours*	2679±910 (4)	5.0±1.5 (4)

* Adult mice at 6 weeks old. Results presented as mean ± SEM. Number of samples in parenthesis.

### Breeding against the MMA knockout mouse model

Breeding the MMA stop codon mouse model against the MMA knockout mouse model [5] (to produce MMA knockout-stop codon mice) also resulted in normal litter sizes, numbers of male and female mice and Mendelian inheritance of the stop condon. MMA stop codon mice with one endogenous *Mut* gene and one *MUT* ^stop^ gene (*Mut* ^+/−^ *MUT* ^Stop+/−^) had double the mRNA expression levels of mice with just one *Mut* gene or one *MUT* ^stop^ gene; however the expressed *MUT* ^stop^ gene would be truncated and inactive. Never the less these mice had normal metabolite and growth patterns (data not presented).

Pups which harbor only the transgene stop codon mutase (no endogenous mouse mutase) (*Mut* ^−/−^ *MUT* ^Stop+/−^) appeared normal at birth, however developed symptoms and died by 24 h in a similar time frame to MMA knockout pups [5]. The MMA knockout-stop codon mouse pup weight did not differ significantly from control pups at birth (1.12±0.15 g, n = 5; compared to control pups 1.19±0.10, n = 59).

These pups were found to have high methylmalonic acid levels in their urine and blood (**Table 1**) correlating with results from MMA knockout pups. The amount of methylmalonic acid in the liver and brain prior to birth was higher than normal; however there was no striking increase soon after birth (**Table 2**), whilst the kidney showed a dramatic increase within the first 16 hours of life.

**Table 2:** Comparison of tissue methylmalonic acid levels of the mouse pups pre- and post-birth.*

Mouse genotype	Age	Liver (nmol/g tissue)	Kidney (nmol/g tissue)	Brain (nmol/g tissue)
**Control pup (** ***Mut*** **^+/+^)**	*1 day prior to birth*	10±0 (5)	10±10 (5)	0±0 (5)
	*0–16 hours after birth*	10±10 (10)	20±20 (9)	10±10 (10)
**MMA Knockout pup (** ***Mut^−/−^*** **)**	*1 day prior to birth*	156±156 (2)	200±200 (2)	255±66 (2)
	*0–16 hours after birth*	5070±3040 (4)	2850±1780 (3)	1190±50 (3)
**MMA Knockout stop codon pup (** ***Mut^−/−^MUT*** **^Stop+/−^)**	*1 day prior to birth*	430±120 (3)	370±490 (3)	130±170 (3)
	*0–16 hours after birth*	670±80 (2)	1470±100 (2)	150±150 (2)
**MMA stop codon rescue pup (** ***Mut^−/−^MUT*** **^Stop+/−^** ***MUT^2h^*** ^**+/−**^ **)**	*1 day prior to birth*	80±10 (2)	20±20 (2)	10±10 (2)
	*0–16 hours after birth*	50±40 (5)	120±20 (4)	30±20 (4)

* Results presented as mean ± SEM. Number of samples in parenthesis.

The MMA knockout-stop codon mouse liver and brain had similar expression levels of the mutase gene to the normal *Mut*, showing that while the gene may be expressed as normal, the protein is truncated and inactive causing problems in the methylmalonyl-CoA metabolic pathway.

### Breeding against the human MMA rescue mouse model

**Figure 4:**
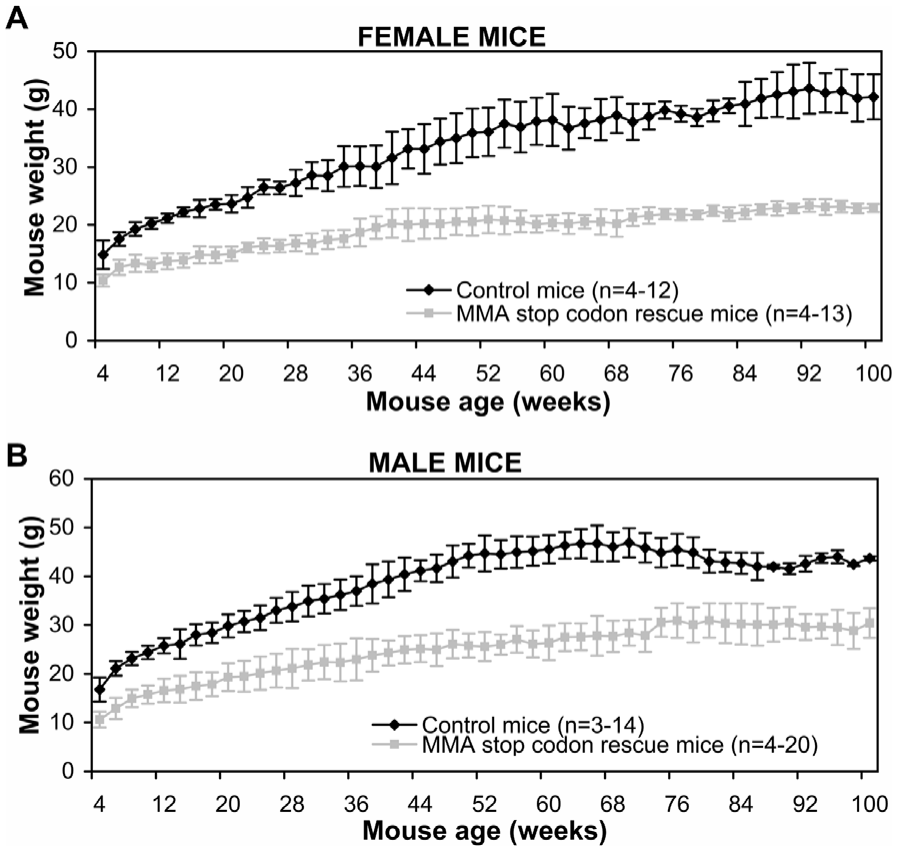
Comparison of weights of control and MMA stop codon rescue mouse lines over 2 years. (A) Female mice (B) Male mice. Data presented as mean ± SEM.

MMA stop codon mice were also bred against mice which harbored the *Mut* knockout gene and the normal human *MUT* gene (MMA rescue mice, *Mut* ^−/−^ *MUT* ^2h+/−^) [6] to produce mice which had no endogenous mutase gene and one copy of each of the normal and stop mutation human mutase genes (‘MMA stop codon rescue mice’, *Mut* ^−/−^ *MUT* ^2h+/−^ *MUT* ^stop+/−^). These MMA stop codon rescue mice weighed significantly less than control mice from 4 weeks old (**Figure 4**). Female mice were about 30% lighter than control litter mates until 10 months, and then the difference increased to about 40%, while male mice were about 30% lighter. Female mouse length ranged from 16% smaller than controls at 6 weeks old to 10% smaller at 2 years old. Male mouse length was also significantly smaller with a range of 19% smaller than controls at 6 weeks old to 9% smaller at 2 years old. The disparity in mouse length was less pronounced than the weight difference, which relates to MMA stop codon rescue mice having little to no abdominal fat deposition.

Within the first 6 months of life MMA stop codon rescue mice were found to have occasional episodes when they appeared sick (decreased movement, hunched over and ruffled fur). The sick mice were provided with a liquid mixture of ground up food pellet and baby formula and placed on a heat pad. They recovered within 2 hours. When provided this diet once per week during the first part of life these episodes were prevented. Heterozygous mice showed none of these health issues.

Comparison of mouse organ weights showed that at 6 months of age the liver of MMA stop codon rescue mice was significantly smaller than control mice (1.06±0.10 g compared to 1.27±0.05 g control mouse liver weight, *p*<0.001, n = 9). When liver weight was compared to body weight (liver weight/whole body weight) the liver size was found to be in proportion to the mouse size. The other organs were not different at this age (kidney, brain, heart, lung, spleen and testis), even though the body weight of MMA stop codon rescue mice was significantly smaller than control mice (18.0±2.2 g compared to 27.2±1.9 g control mouse weight, *p*<0.001, n = 9).

At 2 years of age the liver, kidney, heart, lung, spleen and brain showed no significant difference in size between control and MMA stop codon rescue mice. However the MMA stop codon rescue mice testis were significantly smaller (0.09±0.005 g compared to 0.11±0.002 g control mouse testis weight, *p* = 0.001, n = 4). When the weight of the mice was taken into account (MMA stop codon rescue mice 27.5±3.0 g, control mice 35.1±3.1 g, *p*<0.05, n = 6), there was no significant difference in the testis, kidney, heart, lung, spleen or brain sizes. Whilst the relative weights of MMA stop codon rescue mouse liver (0.054±0.003, compared to control mouse liver 0.043±0.002, n = 5, *p*<0.001) and kidney (0.015±0.001, compared to control mouse kidney 0.012±0.001 n = 5, *p*<0.005) were significantly higher than control mice.

The MMA stop codon rescue mice had increased methylmalonic acid in their blood and tissues (**Tables 1** **–** **3**), however not as high as the MMA knockout-stop codon mice, thus the imbalance in metabolite levels could be partially corrected by addition of human methylmalonyl-CoA mutase enzyme activity via mouse breeding.

**Table 3:** Comparison of tissue methylmalonic acid levels of the mouse models.*

Mouse genotype	Liver (nmol/g tissue)	Kidney (nmol/g tissue)	Brain (nmol/g tissue)	Muscle (nmol/g tissue)
**Control adult (** ***Mut*** **^+/+^)**	4.8±2.0 (15)	9.3±6.2 (27)	6.7±4.1 (34)	2.3±1.1 (10)
**MMA stop codon adult (** ***Mut*** **^+/+^** ***MUT*** **^Stop+/−^)**	4.0±2.0 (6)	10.1±7.2 (6)	7.6±3.8 (6)	2.8±1.5 (6)
**MMA stop codon rescue adult (** ***Mut^−/−^MUT*** **^Stop+/−^** ***MUT^2h^*** ^**+/−**^ **)**	4272±1322 (8)	3492±1015 (7)	206±109 (7)	405±249 (7)

* Mice at 6 weeks old. Results presented as mean ± SEM. Number of samples in parenthesis.

Blood C3 (propionylcarnitine) levels of normal pups did not change with time; however there was an increase in mean concentration in both the MMA knockout-stop codon mouse pups and MMA stop codon rescue pups (**Table 1**). The results from these two new mouse models mirror that of the MMA knockout and MMA rescue pups (data not presented) suggesting that the stop codon carrying *MUT* gene was producing little to no active methylmalonyl-CoA mutase.

**Figure 5:**
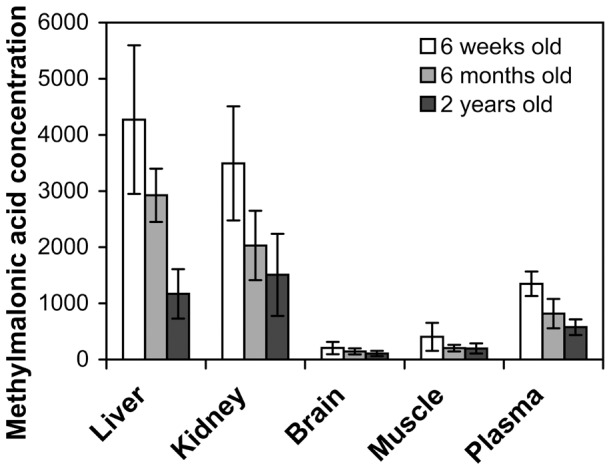
Comparison of tissue and plasma methylmalonic acid levels in the MMA stop codon rescue mice. Tissue results presented as mean µmol/g tissue ± SEM (n = 8). Control samples had less than 15 µmol/g tissue. Plasma results presented as mean concentration (µmol/L) ± SEM (n = 8), control plasma samples had less than 1 µmol/L methylmalonic acid.

Comparing the change in methylmalonic acid with age showed that there was a decrease with age in tissue and plasma levels (**Figure 5**); whilst there was an increase in C3 levels with age (from 9.8±3.0 µmol/L at 3 weeks old, n = 10, to 17.6±5.2 µmol/L at 2-years old, n = 9). Control samples did not vary greatly with age and had an average of 1.23±0.33 µmol/L C3 (n = 55).

Urine methylmalonic acid concentrations in control samples did not vary greatly with age and had an average of less than 150 µmol/mmol creatinine over a 2 year period. MMA stop codon rescue pups had 1130±305 µmol/mmol creatinine (n = 19) at 3 days old, which then rose to 23400±7820 µmol/mmol creatinine (n = 25) at 6 weeks of age and remained consistently high.

## Discussion

A new mouse model which recapitulates the metabolite levels of a patient with methylmalonic aciduria has been developed. The mouse model contains the human methylmalonyl-CoA mutase locus carrying a stop codon mutation identified from a patient with *mut* ^0^ MMA. Mice with only the stop codon gene (no endogenous mouse methylmalonyl-CoA mutase) died within 24 hours of birth with drastically increased urine and blood metabolite levels. This severe phenotype could be partially rescued by breeding the mice to also harbor the normal human methylmalonyl-CoA mutase locus.

Various studies have identified over 100 pathological mutations from patients with *mut* MMA [7]. With the exception of the N219Y mutation, the majority of these are “private” and due to single base nonsense/missense changes [8]. Up to 14% of reported *mut* MMA mutations result from a stop codon [3], [4], [7], [9]. The R403 stop mutation was chosen for a number of reasons: 1) it was identified from our patient population and would therefore be ideal for the translation of gene correction studies into clinical trials; 2) it is predicted to result in a severe phenotype; and 3) it consists of a single base change. For these reasons it is anticipated it will allow the investigation of a number of therapeutic approaches. For example, treatment with aminoglycosides and related compounds to investigate the phenomenon of “read through” to overcome the effects of stop mutations, gene correction using small fragment recombination and gene replacement. Furthermore as this mutation results in a severe phenotype [4], any degree of correction has the potential to be readily detectable.

Microinjection of the modified pBAC_MMA resulted in one transgenic mouse line being produced with an intact locus and single integration site. Surprisingly a high copy number of the transgene was determined. As it is planned to use these mice in gene correction studies this may be beneficial in providing a number of sites to target for correction.

Hemizygous MMA stop codon mice were found to have a normal phenotype and unaltered life span. When this mouse model was bred against the MMA knockout mice, pups with no endogenous mouse mutase gene were produced. The MMA stopcodon knockout pups were not physically different to control pups at birth, however the high metabolite levels soon caused severe health problems leading to death, similar to the problems seen in human cases of MMA.

MMA stopcodon knockout pups have a very similar etiology to the MMA knockout mouse model with high levels of metabolites in blood and tissue, and limited life span. Whist these mouse models are recapitulating the human disorder, and maybe useful for testing *in utero* treatment strategies, generally babies with the disorder are not identified until they are born, therefore it would be more valuable to have a model which can be tested for treatment options once the are pups born. Thus we developed the MMA stop codon rescue mice.

MMA stop codon mice were bred against mice which harbored the *Mut* knockout gene and the normal human *MUT* gene to produce mice which had no endogenous mutase gene and one copy of each of the normal and stop mutation human mutase genes (‘MMA stop codon rescue mice’). In comparison to the MMA rescue mouse model this new MMA stop codon rescue mouse model has very similar phenotypic characteristics, which shows that the stop codon mutation is not producing functional full length methylmalonyl-CoA mutase protein and thus not alleviating the phenotype.

The MMA stop codon rescue mice, similar to the MMA rescue mouse model, is only partially rescued by the human *MUT* gene, suggesting that the human mutase in a mouse background is less efficient (either by reduced protein production or an enzyme catalysis problem) than the native mouse enzyme. The health of the partially rescued mice is initially generally good, but they have episodes where they deteriorate over a couple of days, although can be treated with additional nutrition. This is similar to the MMA rescue mouse model, where the health of the mice is more stable after about 6 months of age.

This MMA stop codon rescue mouse model has significantly higher methylmalonic acid in the urine compared to the blood as organic acids are typically cleared from the bloodstream by the kidney. Soon after birth the amount of methylmalonic acid in the kidney also increases. The effects of high metabolite levels in the urine may cause an enlargement of the kidney in 2-year old mice. The liver was also enlarged at 2 years, whilst other organs were not affected. Human cases of MMA may show an enlarged liver as a symptom as well as long term detrimental effects on the kidney. Many of the severe symptoms of MMA are not recapitulated in this mouse model; however the key biochemical indications are present.

The MMA stop rescue mouse model size and weight gain have been significantly affected, which is a concerning feature in the human disorder. One of the diagnostic characteristics of children with MMA is their failure to thrive, similar to this mouse model. Whilst there was a significant difference in weight between control and MMA stop codon rescue mice, the difference in mouse length was less distinctive. The weight difference can most likely be accounted for by the lack of abdominal fat deposits in the MMA stop codon rescue mice.

Whilst there are mouse models for missense mutations [10], [11], [12], [13], [14], [15] this is the first described model for an inherited metabolic disorder. This MMA stop codon mouse model is important in that it will allow *in vivo* testing of stop codon read through compounds for a human mutation. More importantly the mutation is introduced centrally onto a 170 kb human BAC and is therefore within the context of the flanking human sequence with appropriate human sequence regulatory elements. Due to the intermediate phenotype of the mouse it allows measurement of phenotypic correction (growth and metabolite levels).

This new stop codon carrying mouse model recapitulates the key features of the human MMA disorder at both the phenotypic and genotypic levels, providing an effective tool for the evaluation of *in vivo* and *ex vivo* gene therapy strategies (e.g. gene correction or suppression of stop codon recognition) with direct applicability to human DNA sequences.

## Materials and Methods

### Ethics statement

All procedures involving treatment of mice were performed with ethics approval from the Murdoch Childrens Research Institute Animal Ethics Committee (Projects: A550 and A649). All surgery was performed under isoflurane or avertin anesthesia, and all efforts were made to minimise suffering.

### Production of the construct by two-stage homologous recombination

The R403stop mutation was inserted into the human mutase locus of bacterial artificial chromosome (BAC), RPCI-11-463L20 (pBAC_MMA) by two-stage homologous recombination to form pBAC_MMA*, R403stop. The first stage involved insertion of the I-Sce I/kan^R^ cassette into the R403stop mutation region of pBAC_MMA exon 6.

The I-*Sce* I/kan^R^ resistance cassette was amplified by long range PCR with proof reading Taq (Invitrogen, Carlsbad, CA), from the plasmid pST98-AS/KanF. The primers (Sigma-Aldrich, Australia) used to amplify this cassette (forward 5′-TACTGCAATAGAAGCAATGGCAGCAGTATTTGGAGGGACTCAGTCTTTGCGGTCCGA ACTCTAAACTGCTGA-3′ and reverse 5′-TTGTGAGACATTCCATCATGTAAGAACCTCCC CAAGGATCAGCCACTTTGGGATCTGAGGTTCTTATGGCTCT-3′) incorporated fifty bases of homology to the targeting mutase sequence onto the end of each primer (underlined sequence). The targeting recombination event deleted 100 bp between the homology regions and inserted the 2.1 kb cassette into exon 6 of the mutase locus (**Figure 1**). PCR screening with human *MUT* exon 6 forward (GGATCCCTACAATAATATTGTCC) and reverse (CTTACCTTTAAAGCAGCAT CATA) primers external to the recombination site identified colonies with the correct recombination.

The R403stop mutation was amplified from patient genomic DNA, using primers exon6F 5′-GGATCCCTACAATAATATTGTCC-3′ and exon6R 5′-CTTACCTTTAAAGCAGCAGCAT CATA-3′. In order to amplify only the mutated allele, the 255 bp exon 6 PCR product was sub-cloned into pGemT (Promega Corporation, Madison, WI). A *Taq*I restriction digestion of the exon 6 PCR product from positive colonies enabled determination of a clone containing the R403stop allele. The C→T mutation in exon 6 removed the *TaqI* restriction site, replacing it with a TGA stop codon.

The second stage of homologous recombination was carried out using a 255 bp exon 6 PCR product amplified from the sub-cloned fragment and electroporated into DY380 (pBAC_MMA:I-*Sce* I/Kan^R^) cells. BAC DNA was extracted from the overnight culture, restriction digested (the I*-Sce* I site was utilized as a counter selection to linearize non-recombinants thereby enriching for correct recombinants) and electroporated into DH10B cells. pBAC_MMA*, R403stop positive colonies were sequenced and restriction digested to exclude rearrangements.

### Microinjection

DNA was microinjected at the Walter and Eliza Hall Institute, Transgenic Animal Facility (Parkville, Victoria, Australia). Mice were developed on the C57BL/6 background.

The male founder was bred against C57BL/6 mice producing the mouse colony (MMA stop codon mice). Mice were kept and cared for in the mouse facility at the Murdoch Childrens Research Institute. They were provided irradiated Barastoc pellets (Ridley AgriProducts, Melbourne, VIC, Australia) and tap water *ad libitum*.

### Genotyping

DNA was extracted from a tail tip sample of each mouse and screened. The DNA was firstly amplified using PCR primers for the human *MUT* exon 6 (exon6F and exon6R primers) resulting in a 255 bp product. This product was digested with *Taq*I restriction enzyme. The R403stop mutation removed the restriction site, thus is not digested, whereas a normal human gene produces two bands of 124 and 131 bp.

When breeding the mice against the other MMA mouse models, the genotyping PCR was a multiplex looking for three genes: Exon 3 which amplified the mouse *Mut* gene (forward 5′-CTATTCTGTTGCTTTTATTATTGT-3′, reverse 5′-GAAAAATATAAGTATTTCTGACCAT-3′, 507 bp product); Neo/Kan which amplified the knockout cassette (forward 5′-ATGATTGAACAAGATGGATT-3′, reverse 5′-GCCATGATGGATACTTTCT-3′, 350 bp product); and Exon 12 which amplified the human *MUT* gene (forward 5′-CAGGGTTTTTATAGTCATTA-3′, reverse 5′-CAAGATTCCCATCACAGT-3′, 276 bp product).

### Copy number

The number of copies of the human mutase locus was determined relative to the mouse by PCR of a highly homologous region and comparison of relative intensities. Primers, expF and expR [5], were designed to amplify between two flanking exons and to amplify equally well the mouse and human sequences.

### mRNA expression

The level of mRNA expression from the human transgene relative to the endogenous mouse locus was compared. Real time RT-PCR was performed using the ABi7300 Real Time PCR system with SYBRgreen PCR Master Mix (Applied Biosystems, Foster City, CA). methylmalonyl-CoA mutase cDNA expression was detected using the primers hmMUT-F (5′-TTCTATAAGGACAACATTAAGGCTGGTC-3′) and hmMUT-R (5′-CAATAGCAACTCCAGCCATTCC-3′). Expression was normalized to human beta actin (forward primer 5′-AGGCACCAGGGCGTGAT-3′ and reverse primer 5′-TCGCCCACATAGGAATCCTT-3′). The hmMUT primers bind to the methylmalonyl-CoA mutase cDNA sequence prior to the stop codon mutation. The results were analyzed using the “delta-delta comparative Ct method” and presented as fold change in mRNA levels in cells treated with different compounds, relative to untreated controls.

### Fluorescence in situ hybridization

Metaphase spreads of fibroblast cell lines from the transgenic mice were prepared according to standard procedure. Intact RPCI-11-463L20 BAC was labeled with Spectrum Red-dUTP (Vysis, Abbott Park, IL, USA), using nick translation according to the manufacturer's recommendation. The slides were mounted and examined using an Olympus BX60 epifluorescence microscope. Images were captured using Cytovision software (Applied Imaging Corp., Santa Clara, CA).

### Methylmalonic acid concentration determination

Tissue methylmalonic acid analysis (liver, kidney, brain and muscle) was performed on frozen tissue samples that were weighed precisely, homogenized in water, sonicated and then centrifuged. The supernatant was then mixed with 1 mmol/L internal standard (^2^H3-methylmalonic acid; Cambridge Isotope Laboratories, Andover, MA), protein precipitate removed, before the supernatant was dried and the samples butylated, then neutralized. Samples were run using a 5 µm, Hypersil ODS 2.1×100 mm C18 column (Thermo Scientific, Waltham, MA). The mobile phase consisted of 20% Buffer A (9∶1 water: 0.2 ammonium formate pH 3.5) and 80% Buffer B (9∶1 methanol: 0.2 mol/L ammonium formate pH 3.5) run with a flow rate of 0.2 mL/min. The transitions 231>119 *m/z* and 234>122 *m/z* were used in the selected reaction monitoring mode for butylated methylmalonic acid and ^2^H_3_-methylmalonic acid respectively. Plasma methylmalonic acid analysis was performed in the same manner, without the initial sample preparation steps.

Urine methylmalonic acid levels were measured by direct injection electrospray MSMS as previously described [5]. Creatinine levels were measured for normalization of urine diluteness.

For the measurement of acylcarnitine by tandem mass spectrometry, blood was collected from mice at various time points and spotted onto a card used for newborn screening (Whatman 903 paper) and analyzed by direct injection electrospray MSMS according to standard methods [16].

### Statistical analysis

Data expressed as mean (± SEM). Analyses were performed using a two-sample *t*-test. Statistical significance was accepted at *p*<0.05.
